# Inhibition of retinal neovascularization by siRNA targeting VEGF_165_

**Published:** 2008-10-30

**Authors:** Xiao-bo Xia, Si-qi Xiong, Wei-tao Song, Jie Luo, Yu-ke Wang, Rong-rong Zhou

**Affiliations:** Department of Ophthalmology, Xiangya Hospital, Central South University, Changsha, China

## Abstract

**Purpose:**

To investigate whether vector-based vascular endothelial growth factor 165 (VEGF)_165_ targeted siRNA expression system (pSilencer^siVEGF^) could inhibit VEGF_165_ expression in vitro and suppresses retinal neovascularization in the murine model of oxygen-induced retinopathy.

**Methods:**

pSilencer^siVEGF^, from which siRNA targeting VEGF_165_ could be generated, was constructed and transfected to human umbilical vein endothelial cells. Then the level of VEGF isoforms in cultured cells was measured by RT–PCR and ELISA. Intravitreal injection of pSilencer^siVEGF^ was performed in mice with ischemic retinopathy. Retinal neovascularization was evaluated by angiography using fluorescein-labeled dextran and quantitated histologically. The levels of VEGF_164_, which is equivalent to human VEGF_165_ in murine retinas were determined by RT–PCR and western immunoblotting.

**Results:**

Expression of VEGF_165_ in cultured cells was greatly curtailed by pSilencer^siVEGF^ under both normoxia and hypoxia conditions. However, the other isoforms, VEGF_189_ and VEGF_121_, were expressed to a similar degree regardless of whether pSilencer^siVEGF^ was administered. Based on angiography and histological analysis, retinal neovascularization in the eyes treated with pSilencer^siVEGF^ were significantly reduced compared to the control eyes. Furthermore, the VEGF_164_ levels in the murine retinas were suppressed by pSilencer^siVEGF^.

**Conclusions:**

Retinal neovascularization in the murine model was significantly attenuated by pSilencer^siVEGF^ through decreasing VEGF_164_ levels in the retinas. pSilencer^siVEGF^ seems to be a potential therapeutic tool for ischemic-induced retinal diseases.

## Introduction

Retinal neovascularization, abnormal formation of new vessels from preexisting capillaries in the retina, is a common complication of many ocular diseases, such as advanced diabetic retinopathy, and retinopathy of prematurity. Neovascularization can lead to fibrosis and disruption of delicate tissues required for vision. Laser photocoagulation as conventional treatment is effective in halting the progression of angiogenesis in the short-term. However, it is also destructive to the retinal tissue, leads to immediate and sometimes significant loss of vision, and does not address the underlying angiogenic mechanisms of the disease. Therefore, therapy targeting molecular mechanisms underlying retinal neovascularization may provide better treatment result and fewer detrimental side effect.

Angiogenesis is a complex process, involving multiple gene products expressed by different cell types, all contributing to an integrated sequence of events. However, laboratory studies have demonstrated that vascular endothelial growth factor (VEGF) plays a central role in several retinal vascular diseases. Clinical trials have confirmed the importance of VEGF in disease pathogenesis [1,2]. Consequently, VEGF becomes an optimal target for inhibition of retinal neovascularization. Accumulated data indicate that attenuation of VEGF activity could effectively suppress retinal neovascularization. Recent treatments based on antibody technology have been proven to be efficacious. Lucentis, a anti-VEGF antibody fragment, has been approved as an antiangiogenic drug for the treatment of ocular neovascularization [3]. Although antibodies are effective, they are not efficient. Large amounts of antibodies are needed to suppress the targeted protein, and the inhibitory effects of antibodies are transient unless these high doses are administered repeatedly.

RNA interference (RNAi) is a recently developed technique to silence proteins in a sequence-specific manner by inhibiting mRNA and consequently reducing protein expression. The high efficiency and specificity of RNAi has made it a powerful and widely used tool for gene therapy. The functional mediator of RNAi is a short double strand RNA (dsRNA) oligonucleotide called small interfering RNA (siRNA) [4]. A growing number of investigations are examining the use of siRNA as a candidate therapeutic agent, Currently, there are two siRNA-based molecules: Cand5, which is a siRNA against all isoforms of VEGF, and siRNA-027, a kind of siRNA targeting VEGF receptor 1 [5]. Acuity Pharmaceuticals (Philadelphia, PA) has begun a Phase II clinical trial for Cand5, and Sirna Therapeutics (San Francisco, CA) is on a Phase 1 clinical trial for siRNA-027. However, due to differential pre-mRNA splicing, a single VEGF gene gives rise to many different VEGF isoforms. To date, five isoforms of human VEGF have been identified: VEGF_121_, VEGF_145_, VEGF_165_, VEGF_189_, and VEGF_206_ [6]. Although VEGF is highly conserved throughout evolution, the murine homologs contain one fewer amino acid. The murine designation for the human VEGF_165_ is VEGF_164_. Of the various isoforms, VEGF_165_ (VEGF_164_) appears to be the major pathological VEGF isoform in the eye [7]. Because VEGF_165_ is a major disease-causing isoform in models of neovascular eye disease, we expected to identify whether retinal angiogenesis could be attenuated by siRNA targeting VEGF_165_.

In this report, we used a vector-based siRNA expression system, which overcomes the limitations of transience and high cost in synthetic siRNAs, to specifically inhibit VEGF_165_ expression in the murine model of proliferative retinopathy. Our data confirm the potential VEGF_165_ inhibitors for the treatment of ocular angiogenesis.

## Methods

### Recombinant pSilencer^siVEGF^ construction

The cDNA oligonucleotides targeting VEGF_165_ mRNA were designed and examined by Guan et al. [13]. A pair of 63 nucleotide oligos containing endonuclease restriction sites at both ends was synthesized by the Sangong Company (Shanghai, China). The sequences used were: First strand-5′–GAT CCG ATA GAG CAA GAC AAG AAA TTC AAG AGA TTT CTT GTC TTG CTC TAT CTT TTT TGG AAA–3′; Second strand-5′–AGC TTT TCC AAA AAA GAT AGA GCA AGA CAA GAA ATC TTT GAA TTT CTT GTC TTG CTC TAT CG–3′ (complementary sequence is indicated in red). The annealed dsDNA oligonucleotides were ligated between the BamHI and HindIII sites on the pSilencer2.1-U6 hygro vector. The targeted VEGF_165_ sequences were GAT AGA GCA AGA CAA GAA A. All inserted sequences were confirmed by DNA sequencing.

### Cell culture and transfection of cells

Human umbilical vein endothelial cell lines (HUVECs) were purchased from the American Type Culture Collection (Manassas, VA). HUVECs were grown in F12K medium, containing 0.1 mg/ml heparin, 20% fetal bovine serum, 0.03 mg/ml endothelial cell growth supplement (BD Biosciences, San Jose, CA), and antibiotic mixtures of 100 U/ml penicillin G and 100 μg/ml streptomycin sulfate. Cells were cultured in an incubator at 37 °C in an atmosphere of 95% air and 5% CO_2_. In the vitro studies, an oxygen concentration of 20% was considered normoxic. Hypoxia was 1% oxygen. In the vivo studies, normoxic mice were raised in room-air-raised. For hyperoxia, mice were kept in 75±2% oxygen. Transfection reagent lipofectamine2000 was used to transfer the pSilencer^siVEGF^ to the HUVECs according to the manufacturer’s protocol (Invitrogen, Carlsbad, CA). In brief, 2×10^5^ HUVECs cells were seeded into six-well plates. A 2.5:1 ratio of lipofectamine–pSilencer^siVEGF^ (Invitrogen) complexes were prepared and added to the HUVECs.

### Reverse transcription and polymerase chain reaction

Whole RNA of cultured cells or murine retinas were isolated with TRIzol® reagent (Invitrogen). Total RNA was isolated using Trizol reagents (Gibco-BRL Life Technologies, Gibco, Carlsbad, CA), followed by treatment for 45 min with RNase-free DNase at 37 °C (Message Clean; GeneHunter Corp., Nashville, TN), phenol:chloroform (ratio 1:1) extraction, ethanol precipitation, and stabilizing in DEPC-treated water. RNA concentration was determined by spectrophotometric readings at 260 and 280 nm. Each 2 mg of RNA extract was reverse-transcribed into cDNA with RevertAidTM First Strand cDNA Synthesis Kit (MBI, Burlington, MD). PCR was performed in 50 µl of a solution containing Taq DNA polymerase, dNTP, VEGF primer, or β-actin primer and RT products. The primer pair, 5′-CGA AGT GGT GAA GTT CAT GGA TG-3′ (sense) and 5′-TTC TGT ATC AGT CTT TCC TGG TGA G-3′ (antisense), was used to amplify human VEGF isoforms in cultured cells. The primer pair, 5′-CCT CCG AAA CCA TGA ACT TTC TGC TC-3′ (sense) and 5′-CAG CCT GGC TCA CCG CCT TGG CTT-3′ (antisense), was designed to yield the PCR products of murine VEGF isoforms. The primer pair, 5′ CGT TGA CAT CCG TAA AGA C 3′ (sense) and 5′ TGG AAG GTG GAC AGT GAG 3′ (antisense), was used to obtain the PCR product of β-actin. After a preincubation for 5 min at 94 °C, 30 cycles of amplification (94 °C for 45 s, 60 °C for 45 s, and 72 °C for 45 s) were performed, and β-actin was used as an internal control. Amplified products were run in a 1.5% ethidium bromide agarose gel, band intensities were captured with a ChemiDoc system and LabWorks software (UVP, Upland, CA), and values were transferred to an Excel spreadsheet for calculation of means and standard errors.

### ELISA assay for VEGF production

The medium from normoxia and hypoxia cultured cells was collected, centrifuged at 750×g for 5 min, and stored at −70 °C. Concurrently, cells were trypsinized and counted. The concentration of the secreted VEGF_165_ isoform in the media was determined with an ELISA kit (R&D Systems, Minneapolis, MN) according to the manufacturer’s instructions and was expressed as pg VEGF/10^5^ cells.

### Animal model of proliferative retinopathy

The study protocol conformed to the ARVO Statement for the Use of Animals in Ophthalmic and Vision Research. The reproducible murine model of oxygen-induced retinopathy (OIR) has been described previously [8]. C57BL/6J mice were bought from Experimental Animal Center of Central South University, Changsha, Hunan province, China. The mice were exposed to less than 300 lx of 12 h cyclical broad spectrum light. The oxygen-treated mice were housed in an incubator. Oxygen concentration was monitored with a Beckman oxygen analyzer (Model D2; Beckman,. Irvine, CA). The cage temperature was maintained at 23 °C±2 °C. The mice were placed in the oxygen chamber with enough food and water to sustain them for 5 days. Postnatal day 7 (P7) C57BL/6J mice and their nursing mothers were exposed to 75%±2% oxygen for five days. On P12, the mice were removed from the chamber and maintained in room air until P17. Mice of the same strain and of the same age were kept in room air and used as control subjects. Intravitreal injections of liposome–pSilencer^siVEGF^ complexes were performed at P12 as described in the next section. At P17, the mice were sacrificed by cardiac perfusion of 4% paraformaldehyde in phosphate-buffered saline (PBS) catalog number of PBS is 20012043 (Gibco), and their eyes were enucleated for RT–PCR and immunohistologic analysis. Alternatively, some mice underwent retinal fluorescein angiography, as described in the next section.

### Intravitreal injections

At P12, mouse pups were deeply anesthetized with a 30 mg/kg intraperitoneal injection of sodium pentobarbital. The lid fissure was opened with a no. 11 scalpel blade, and the eye was proptosed. Intravitreal injections were performed by first entering the eye with an Ethicon TG140–8 suture needle at the posterior limbus. A 32-gauge Hamilton needle and syringe were used to deliver 1 µl liposome-pSilencer^siVEGF^ complex (0.5 µl liposome and 1–1000 ng of pSilencer^siVEGF^) or 1 µl control complex (0.5 µl liposome and 0.5 µl of 2 mg/ml pSilencer null vector) into the vitreous cavity.

### Angiography with high-molecular-weight fluorescein-dextran

At P17, mice were anesthetized with a 30 mg/kg intraperitoneal injection of sodium pentobarbital sodium, and cardiac perfusion was performed with 1 ml PBS containing 50 mg/ml fluorescein-labeled dextran (2×10^6^ average molecular weight; Sigma, St Louis, MO), clarified by centrifugation for 5 min at 10,600 xg. Subsequently, the mice were sacrificed, and their eyes were enucleated. The retinas were dissected and flatmounted on microscope slides with glycerol gelatin.

### Histological analysis of neovascularization

The eyes of sacrificed P17 mice were enucleated, fixed overnight with 4% paraformaldehyde in PBS, and embedded in paraffin. Half of the group of mice were used for angiography with high-molecular-weight fluorescein-dextran, and the other half were used for histological analysis of neovascularization. Paraffin-embedded axial serial sections of the retina, 6 µm thick, were obtained starting at the optic nerve head. After staining with periodic acid-Schiff reagent and hematoxylin, we evaluated 10 intact sections of equal length, each 30 µm apart, for a span of 300 µm. All retinal vascular cell nuclei anterior to the internal limiting membrane were counted in each section according to a masked protocol. The mean of all ten counted sections yielded average neovascular cell nuclei per 6μm section per eye.

### Western blot analysis of VEGF in the murine retina

The murine retinas were collected and lysed in lysis buffer composed of 150 mmol/l NaCl, 50 mmol/l Tris-HCl, pH 7.4, 2 mmol/l EDTA, and 1% NP-40 that also contained protease inhibitors (Boehringer Mannheim, Mannheim, Germany). Total protein (30 μg per lane) was loaded on SDS-polyacrylamide gel and transferred onto a nitrocellulose membrane and incubated with a 1:500 dilution rat monoclonal anti-VEGF antibody Santa Cruz (Santa Cruz, CA, catalog number sc-80436) and a 1:10,000 dilution mouse monoclonal anti-β-actin antibody (Sigma, St. Louis, MO), followed by incubation with corresponding secondary antibodies (peroxidase conjugated). The enhanced chemiluminescence (ECL) chemiluminescence reagent was added to the membrane according to the manufacturer’s directions. The membrane was exposed to an X-ray film for 1 min before it was developed and fixed. Images of blots were entered into a computer using a scanner (Epson, Nagano, Japan) and analyzed using LabWorks Software (UVP). Then, values were transferred to an Excel spreadsheet for calculation of means and standard errors.

### Statistics

The results were expressed as mean±standard error of mean (SEM). One-way ANOVA followed by the least significant difference (LSD)-*t*-test were used to evaluate significant differences. A p value <0.05 was considered statistically significant.

## Results

### Suppression of VEGF_165_ expression in cultured cells by pSilencer^siVEGF^

**Figure 1:**
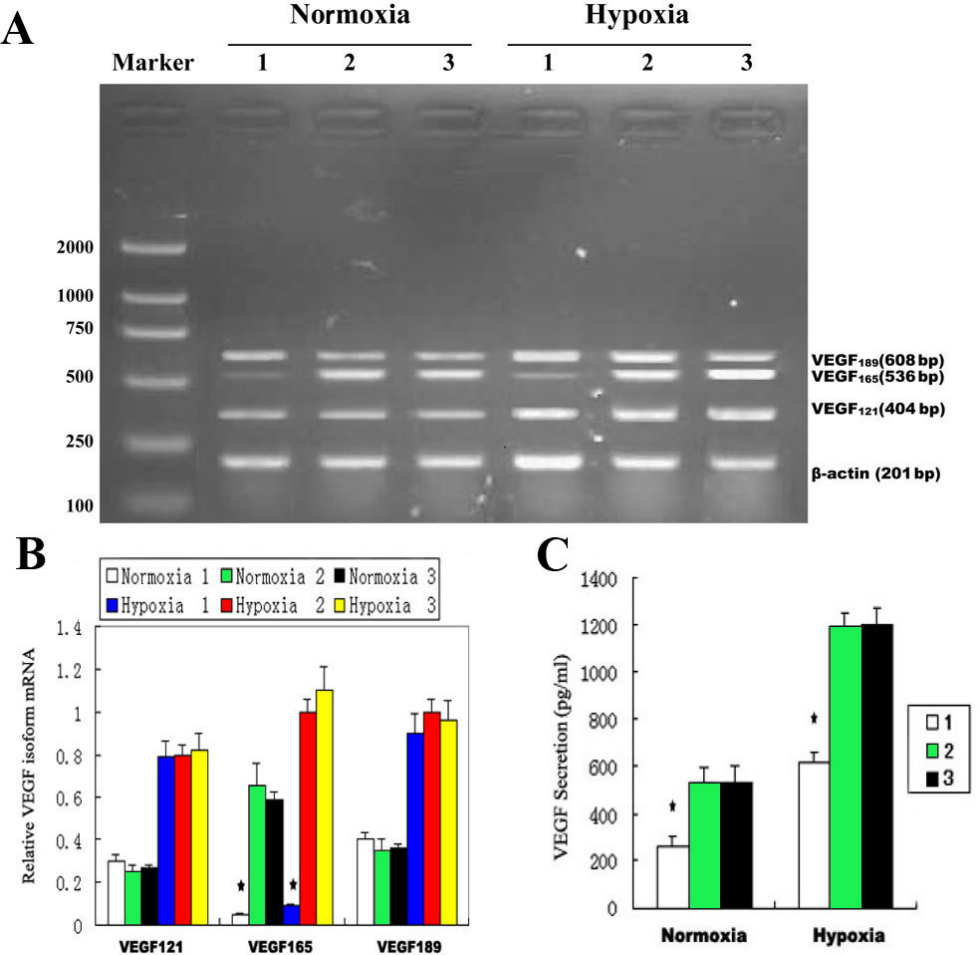
Inhibition of VEGF_165_ expression in cultured cells by pSilencer^siVEGF^ **A:** Shown is a gel image of a PCR product of VEGF isoforms. Vascular endothelial growth factor (VEGF)_121_, VEGF_165_, and VEGF_189_ mRNA levels were determined by RT–PCR. The lanes are marked as follows: 1) pSilencer^siVEGF^-transfected cells; 2) pSilencer null vector-transfected cells; and 3) nontransfected cells. **B:** The levels of VEGF isoforms were quantitated by densitometry and normalized by β-actin mRNA levels. **C**: VEGF_165_ levels in the conditioned media were measured by ELISA kits and normalized to cell counts (1×10^5^). In a comparison with control cells, hypoxia-induced VEGF_165_ mRNA and secreted VEGF_165_ expression were found to be decreased after transfection with pSilencer^siVEGF^, while VEGF_121_ and VEGF_189_ were expressed to a similar degree regardless of whether pSilencer^siVEGF^ was administered. Asterisk indicates p<0.05 compared to the control cells.

To determine whether vector-based VEGF_165_ targeted siRNA expression system (pSilencer^siVEGF^) could suppress the expression of VEGF_165_, we transfected pSilencer^siVEGF^ plasmid to HUVECs. Then the transfected cells were further cultured under normoxia (20% O_2_) and hypoxia (1% O_2_) conditions for 24 h, together with null vector transfected and non-transfected HUVECs (as control). All these cells were later used for RT–PCR and ELISA analysis. As shown in Figure 1A,B, VEGF_165_ mRNA levels in pSilencer^siVEGF^ transfected cells were dramatically decreased under both normoxic and hypoxic conditions compared to the control cells (p<0.05), while VEGF_121_ and VEGF_189_ were expressed to a similar degree regardless of whether pSilencer^siVEGF^ was applied. Correspondingly, the amount of VEGF_165_ protein secreted into the medium of cultured cells was greatly inhibited after pSilencer^siVEGF^ transfection (Figure 1C), indicating that pSilencer^siVEGF^ could specifically decrease VEGF_165_ expression in cultured cells without alteration of other VEGF isoforms expression under both normoxia and hypoxia conditions.

### Angiographic evaluation of the effect of pSilencer^siVEGF^ on retinal neovascularization

**Figure 2:**
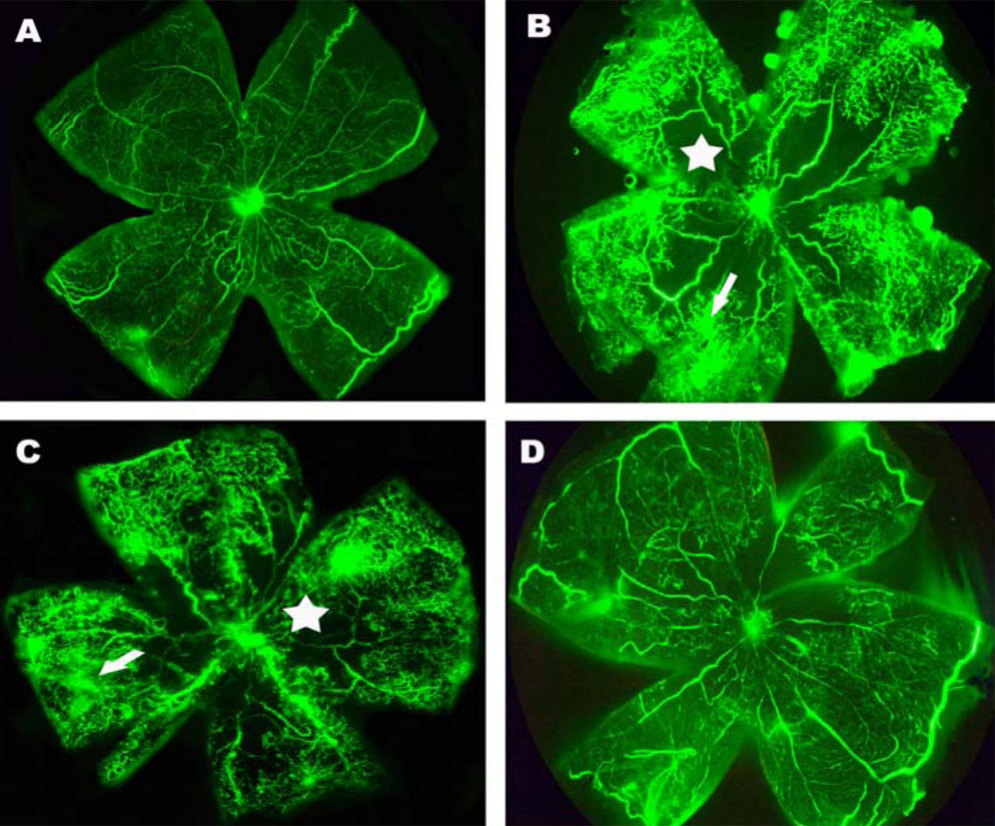
Angiographic analysis of the effect of pSilencer^siVEGF^ on the murine model of retinal neovascularization. Retinal flatmounts were examined by fluorescein dextran angiography. **A**: Shown is a retinal flatmount of mice raised in room air. **B**: In this retinal flatmount of mice exposed to hyperoxia, neovascular tufts appear as hyperfluorescence at the junction between the perfused and the nonperfused area. Arrow points to retinal neovascularization, and star marks an area of nonperfusion. **C**: In this retinal flatmount of hyperoxia exposed mice injected with pSilencer null vector, retinal neovascularization and nonperfusion area were not attenuated. **D**: Shown is a retinal flatmount of hyperoxia-exposed mice injected with pSilencer^siVEGF^. The amount of neovascular tufts was markedly reduced and the area of nonperfusion was diminished.

Injection of liposome –pSilencer^siVEGF^ complexes was performed at P12. Injection of fluorescein-labeled dextran into left ventricle was done at P17, followed by retina flatmount. The retinas of room air-raised mice revealed a normal capillary network without nonperfusion area and neovascular tufts. However, retinas from hyperoxia-exposed mice with pSilencer null vector injection or without any injection contained multiple neovascular tufts and central nonperfusion areas. In contrast, fewer neovascular complexes could be found in retinas of mouse eyes injected with pSilencer^siVEGF^ (Figure 2).

### Histological analysis of retinal neovascularization

**Figure 3:**
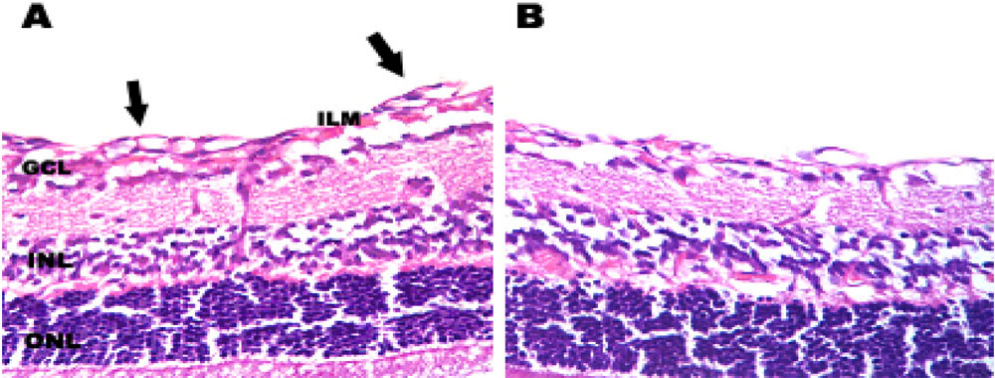
Histological analysis of the effects of treatment with pSilencer^siVEGF^ on ischemia-induced retinal neovascularization. **A:** Postnatal day 17 (P17) retina from the eye of a hypoxic mouse injected with 1000 ng pSilencer null vector. Extensive preretinal neovascular loops are apparent (black arrows). **B:** P17 retina from the eye of hypoxic mouse injected with 1000 ng pSilencer^siVEGF^. Preretinal neovascular loops were not as apparent, suggesting that treatment reduced preretinal neovascularization compared with the pSilencer null vector-injected eye. The following abbreviations were used in the figure: interlimiting membrane (ILM), ganglion cell layer (GCL), inner nuclear layer (INL), and outer nuclear layer (ONL).

**Figure 4:**
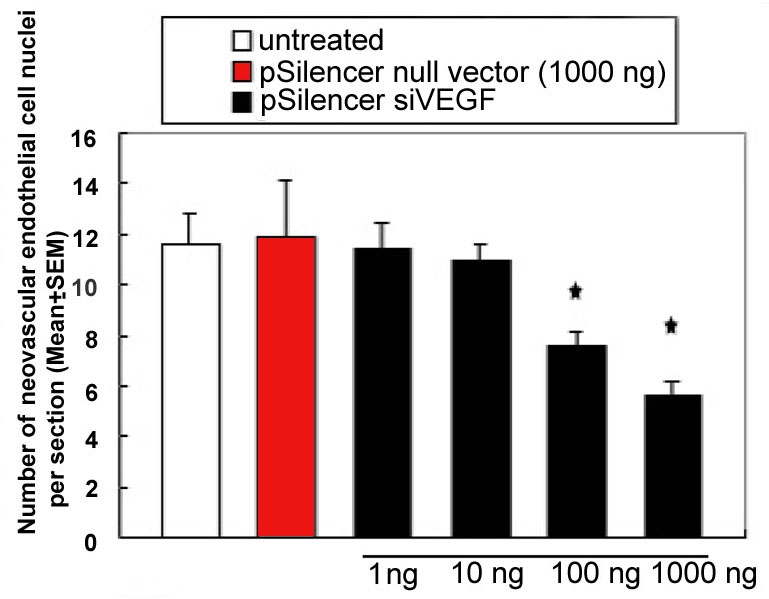
Dose-dependent inhibition of retinal neovascularization by pSilencer^siVEGF^ Administration of pSilencer^siVEGF^ at doses 1–1000 ng to hypoxic mice resulted in a dose-dependent inhibition of retinal neovascularization as measured by counting the number of endothelial cell nuclei per section, with a maximum inhibitory effect of 50% at the 1000 ng dose. Asterisk (*) indicates p<0.05 compared to the control eye.

Neovascularization was assessed histologically by counting the endothelial cell nuclei anterior to the inner limiting membrane. Retinas from hyperoxia-exposed mice with injection of 1000 ng pSilencer null vector or without any injection (OIR model) were found to contain multiple neovascular tufts extending into the vitreous. In contrast, fewer neovascular complexes were observed in pSilencer^siVEGF^-injected retinas (Figure 3). Administration of 1–1000 ng doses of pSilencer^siVEGF^ resulted in a dose-dependent decrease in endothelial cell nuclei numbers anterior to the inner limiting membrane (i.e., inhibition of retinal neovascularization), with a maximum inhibitory effect of 50% at the 1000 ng dose (Figure 4).

### Inhibition of VEGF_164_ expression in murine retinas by pSilencer^siVEGF^

**Figure 5:**
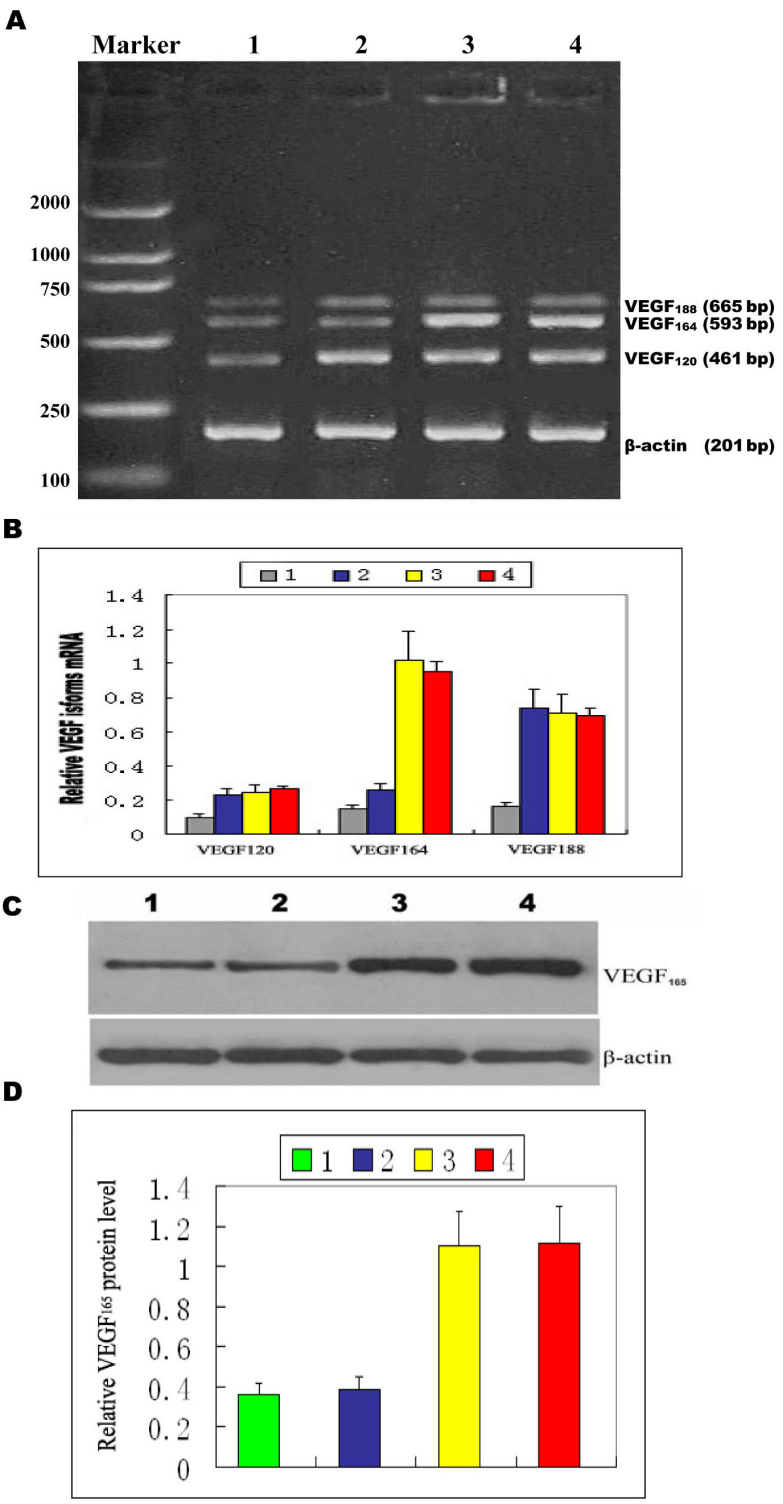
Suppression of vascular endothelial growth factor expression in murine retinas by pSilencer^siVEGF.^ Murine retinas were extracted and examined for VEGF level. One-way ANOVA followed by the LSD-*t*-test were used to evaluate significant differences. A p value <0.05 was considered statistically significant. Error bars are standard error of mean (SEM). **A**: VEGF_120_, VEGF_164_, and VEGF_188_ mRNA levels in murine retinas were examined by RT–PCR. VEGF_164_ mRNA in murine retinas was downregulated after intravitreal administration of pSilencer^siVEGF^, whereas VEGF_120_ and VEGF_188_ levels in murine retinas remained unchanged. Lanes are marked as follows: 1) room air-raised mice; 2) murine model with pSilencer^siVEGF^ injection; 3) murine model with pSilencer null vector injection; and 4) murine model of oxygen-induced retinopathy. **B:** Relative VEGF isoforms mRNA quantification was related to β-actin mRNA. **C**: Shown is an immunoblot assay for VEGF_165_ protein in the murine retinas. The total amount of VEGF_164_ immunosignal in murine retinas was reduced by pSilencer^siVEGF^. The following abbreviations are used in panel **C**: ganglion cell layer (GCL), inner nuclear layer (INL), and outer nuclear layer (ONL). **D:** The levels of VEGF_165_ were quantitated by densitometry and normalized to β-actin protein levels. Data are expressed as mean±SEM and were analyzed by one-way ANOVA followed by LSD-*t*-test. The means of groups 3 and 4 are statistically greater than the means of groups 1 and 2 (p<0.05).

To determine whether intravitreal injection of pSilencer^siVEGF^ could decrease VEGF_164_ expression in the murine retinas, we extracted retinas and assessed them by RT–PCR and immunohistochemical analysis. Some retinas were used for RNA extraction and RT–PCR analysis, the others were embedded in paraffin and examined by immunohistochemistry. Figure 5A,B show the PCR products of VEGF isoforms in the murine retinas. Compared to the control eyes, the level of VEGF_164_ mRNA was significantly downregulated in the retinas of hypoxic animals treated with pSilencer^siVEGF^, whereas VEGF_120_ and VEGF_188_ levels in the murine retinas remained unchanged after local administration of pSilencer^siVEGF^. To confirm the effect of pSilencer^siVEGF^ on the expression level of VEGF_164_ at the protein level, we performed immunohistochemical studies to investigate VEGF_164_ expression patterns in the retinas of the murine model of OIR. We performed western immunoblotting to assess the VEGF164 protein level in the retinas of the murine model of OIR. As shown in Figure 5C,D, local administration of pSilencer^siVEGF^ significantly decreased expression of VEGF protein in the murine retinas.

## Discussion

Vascular homeostasis is regulated by two counter-balancing systems: angiogenic stimulators and angiogenic inhibitors [9,10]. Under pathological conditions, such as diabetic retinopathy and retinopathy of prematurity, the retina increases the production of angiogenic stimulators and reduces the production of angiogenic inhibitors, which would disrupt the balance between the positive and negative regulators of angiogenesis [9]. VEGF as major angiogenic stimulator is upregulated in ocular neovascular diseases [11]. However, due to alternative splicing of mRNA, VEGF has multiple isoforms. VEGF_165_ (VEGF_164_ in mice) is the major disease-causing isoform in models of neovascular eye disease. VEGF_165_ was found to be more potent than other VEGF isoforms in inducing vascular leakage and breakdown of blood–retinal barrier [12]. Hence, VEGF_165_ was selected for this study as a target for suppressing retinal neovascularization. We hoped that therapy targeting VEGF_165_ in the retina could increase specificity and efficacy of the treatment.

To decrease pathological VEGF_165_ upregulation, we constructed a vector-based VEGF_165_ targeted siRNA expression system (pSilencer^siVEGF^) for our studies. pSilencer^siVEGF^ has been shown to specifically inhibit VEGF_165_ expression in normoxia-cultured tumor cells [13]. It has been hypothesized that sustained overproduction of VEGF by hypoxia retinal cells promotes retinal neovascularization in several neovascular eye diseases [1]. Consequently, close attention was paid to whether pSilencer^siVEGF^ could work properly under hypoxia conditions. VEGF_165_ was found to be decreased dramatically not only in the hypoxia cultured cells but also in the hypoxia retinal cells in the murine animal models, suggesting that hypoxia-induced VEGF could be abrogated by pSilencer^siVEGF^ under hypoxia conditions. We surmise that overproduction of VEGF by hypoxia retinal cells may also be blocked by pSilencer^siVEGF^ in human neovascular eye diseases such as diabetic retinopathy.

In the current study, retinal neovascularization in the murine model was greatly inhibited by 1000 ng of pSilencer^siVEGF^. This dramatic decrease in retinal neovascularization could be attributed to the following reasons. First, the localization of plasmids mediated by cationic liposome is close to the site of hypoxia-induced VEGF generation. VEGF_164_ level in the retinas of the murine model was found to increase in the inner nuclear layer and ganglion cell layer in this study, which was consistent with a previous study [14]. However, cationic liposome has been confirmed as an effective reagent for retinal gene transfer, which could carry plasmids into retinal ganglion cells and retinal pigment epithelial cells through the injection of plasmids and liposome complex into the vitreous [15]. Accordingly, intravitreal injections of pSilencer^siVEGF^ could be successfully carried into retinal cells, which are in charge of producing hypoxia-induced VEGF by liposome. Second, the catalytic nature of RNAi entered the retinal cells. Small hairpin RNA (shRNA) targeting VEGF_165_ could be generated and processed into siRNA in these cells. The siRNA targeting VEGF_165_ binds to the RNA induced silencing complex (RISC), which in turn becomes activated. The activated RISC complex seeks out the VEGF_165_ mRNA and then splices the mRNA at the site of the homologous sequence [4]. Furthermore, in multiple turnover kinetic fashion, the activated RISC can seek another VEGF_165_ mRNA to bind and destroy. One activated RISC complex can bind and destroy hundreds of VEGF_165_ mRNA. Finally, the high efficiency of pSilencer^siVEGF^ is attributed to the divisional rate of the retinal cells. The division rate of cells that have taken in siRNA is an important factor when determining durability of silencing in cells [16], which leads to dilution of the activated RISC as it is divided between daughter cells. Because the division rate of retinal cells is slow, the silencing effect in the cells could last until upregulation of VEGF in ischemic retina disappears.

In summary, we found that siRNA targeting VEGF_165_ expressed from pSilencer^siVEGF^ could decrease VEGF_165_ expression in vitro and reduce retinal neovascularization in a murine model of OIR. However, further experiments are necessary to determine whether detrimental side effects will appear after local administration of pSilencer^siVEGF^.
